# Biodegradable, Super-Strong, and Conductive Cellulose Macrofibers for Fabric-Based Triboelectric Nanogenerator

**DOI:** 10.1007/s40820-022-00858-w

**Published:** 2022-04-28

**Authors:** Sanming Hu, Jing Han, Zhijun Shi, Kun Chen, Nuo Xu, Yifei Wang, Ruizhu Zheng, Yongzhen Tao, Qijun Sun, Zhong Lin Wang, Guang Yang

**Affiliations:** 1 College of Life Science and Technology, Huazhong University of Science and Technology, Wuhan, 430074 People’s Republic of China; 2 Beijing Institute of Nanoenergy and Nanosystems, Chinese Academy of Sciences, Beijing, 101400 People’s Republic of China; 3 School of Nanoscience and Technology, University of Chinese Academy of Sciences, Beijing, 100049 People’s Republic of China; 4 Center On Nanoenergy Research, School of Physical Science and Technology, Guangxi University, Nanning, 530004 People’s Republic of China; 5 School of Materials Science and Engineering, Georgia Institute of Technology, Atlanta, GA 30332-0245 USA; 6 State Key Laboratory of New Textile Materials and Advanced Processing Technologies, Wuhan Textile University, Wuhan, 430200 People’s Republic of China

**Keywords:** Biodegradable, Conductive macrofiber, Fabric-based TENG, Energy harvesting, Self-powered sensors

## Abstract

Electronic fibers used to fabricate wearable triboelectric nanogenerator (TENG) for harvesting human mechanical energy have been extensively explored. However, little attention is paid to their mutual advantages of environmental friendliness, mechanical properties, and stability. Here, we report a super-strong, biodegradable, and washable cellulose-based conductive macrofibers, which is prepared by wet-stretching and wet-twisting bacterial cellulose hydrogel incorporated with carbon nanotubes and polypyrrole. The cellulose-based conductive macrofibers possess high tensile strength of 449 MPa (able to lift 2 kg weights), good electrical conductivity (~ 5.32 S cm^−1^), and excellent stability (Tensile strength and conductivity only decrease by 6.7% and 8.1% after immersing in water for 1 day). The degradation experiment demonstrates macrofibers can be degraded within 108 h in the cellulase solution. The designed fabric-based TENG from the cellulose-base conductive macrofibers shows a maximum open-circuit voltage of 170 V, short-circuit current of 0.8 µA, and output power at 352 μW, which is capable of powering the commercial electronics by charging the capacitors. More importantly, the fabric-based TENGs can be attached to the human body and work as self-powered sensors to effectively monitor human motions. This study suggests the potential of biodegradable, super-strong, and washable conductive cellulose-based fiber for designing eco-friendly fabric-based TENG for energy harvesting and biomechanical monitoring.

**Supplementary Information:**

The online version contains supplementary material available at 10.1007/s40820-022-00858-w.

## Highlights

- The cellulosed-based macrofibers possess super-strong tensile strength of 449 MPa and excellent electrical conductivity of 5.32 S cm^−1^.
- The cellulosed-based macrofiber can be degraded within 108 h in the cellulase solution.
- The designed fabric-based triboelectric nanogenerator (TENG) shows a maximum output power of 352 μW, which can effectively drive commercial electronics.
- The designed fabric-based TENG as self-powered sensors can effectively monitor the human movement of walking, running, jumping, arm lifting, arm bending, and leg lifting.

**Supplementary Information:**

The online version contains supplementary material available at 10.1007/s40820-022-00858-w.

## Graphical abstract

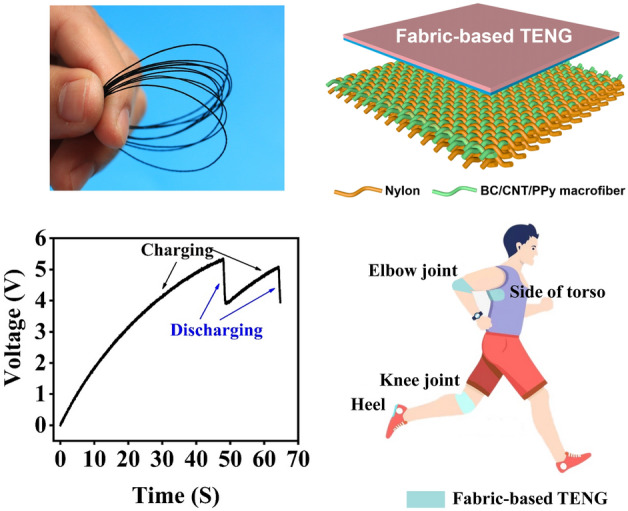

## Introduction

Fabric-based triboelectric nanogenerator (TENG) can easily convert human mechanical energy into electricity [1–4]. With the advantages of lightweight, flexibility, breathability, and washability, it has become an effective power source for wearable commercial electronics. The fabric-based TENG is driven by the Maxwell’s displacement current, typically operating in four different modes, i.e., vertical contact-separation mode, lateral-sliding mode, single electrode mode, and freestanding mode [5–8]. Regarding structures and fabrication methods, fabric-based TENG mainly covers single fiber structure, 2D textile forming structure, 3D textile forming structure and multilayer fabric stacking structure [9–12]. To achieve sophisticated fabric-based TENG capable of satisfying versatile applications, elaborate preparation of robust conductive materials is of great significance, which needs to satisfy good mechanical properties, environment stability and reliable safety.

Conductive materials for the electrodes of fabric-based TENG can be mainly categorized into five types, including metals and its derivatives, carbonaceous fillers, conductive polymers (CPs), liquid electrode, and hybrid conductive fillers. For metals and metallic derivatives (such as Cu, Ag, Ni, Al, Au nanowires and nanoparticles, or stainless-steel rods) [13–18], Dong et al. utilized Ag conductive yarn as electrode and wrapped it with polytetrafluoroethylene (PTFE) and nylon66 (PA66) for knitting a stretchable and comfortable fabric-based TENG [19]. For carbonaceous fillers (such as carbon nanotubes (CNTs) and graphene oxide (GO), carbon fiber) [20–22], researchers have twisted the CNTs coated cotton yarn and further coated it with PTFE to fabricate a fiber-based TENG, which can be integrated into a common cloth system and trigger a wireless body temperature sensor system [23]. For conductive polymers (such as polypyrrole (PPy), polyaniline (PANI), and poly(3,4-ethylenedioxythiophene:poly(styrenesulfonate) (PEDOT:PSS)) [24, 25], Lee et al. utilized PEDOT:PSS coated textile as both electrode and friction layer to design a fabric-based TENG, which possesses a large strain sensing range from 10 to 160%, and can effectively detect human activities such as standing up, walking, running, arm bending, a sudden fall, and sitting [26]. For liquid electrode (such as Ga-indium (EGaIn) and potassium iodide and glycerol (KI-Gly)) [27], Yang et al. developed a liquid–metal-based TENG (LM-TENG) by employing EGaIn as the electrode and silicon rubber as the friction layer. The LM-TENG can maintain stable performance under various deformations (such as stretching, folding, and twisting) and is capable of harvesting energy from human walking, arm shaking and hand patting to sustainably drive wearable electronics devices [28]. For hybrid conductive fillers (such as PPy/CNT, Ag NWs/CNT, and PEDOT:PSS/Ag NWs) [29–31], Liu et al. utilized PTFE coated Ag and aligned CNTs sheets as the electrodes to fabricate fabric-based TENG [32]. The designed fabric-based TENG can efficiently convert mechanical energy into electrical energy with an open-circuit voltage over 500 V and a maximal power density of 153.8 mW m^−2^. Table S1 compares the triboelectric output response and the applications of fabric-based TENGs with various conductive materials as electrodes. In spite that the output performance of fabric-based TENG based various electrodes has been extensively improved, few reports have concerned on the biodegradability of electrodes without scarifying its conductivity and mechanical properties.

Note that many degradable or green materials have been used to fabricate TENG, such as PLGA, starch, chitosan, silk, plant protein, laver, and BC [33–42]. However, these materials are only used as the friction layer of TENG in the form of membranes (Table S2), few works were reported conductive fibers based on these materials for fabricating fabric-based TENG. Notably, cellulose, a well-known natural polymer, is renewable, biodegradable, and enriched in nature [43]. Specifically, cellulose produced by bacteria (i.e., bacterial cellulose, BC), is also an abundant natural polymer with three-dimensional (3D) network composed of nanofibers. The 3D structures endow BC excellent mechanical properties, good biocompatibility/biodegradability, high porosity, and high purity [44–46]. BC has been extensively utilized in the field of flexible electronics, biomedical devices, cosmetics, tissue engineering, drug release, and wound dressing [47], exhibiting its excellent safety and reliability. To explore more applications based on conductive BC, the CNTs and PPy with outstanding mechanical, thermal, chemical and structural properties are good options (as conductive fillers) to improve the conductivity of BC composites [48–52]. Thus, how to further prepare fibrous conductive BC is critical to construct eco-friendly and biodegradable fabric-based TENG toward wearable energy harvesting and self-powered sensing.

Here, we report a biodegradable and super-strong conductive macrofiber via wet-stretching and wet-twisting strip-shaped BC incorporated with conductive CNTs and PPy. CNTs are incorporated into the BC network with surfactant in the form of physical doping, while PPy is synthesized by in-situ oxidation under iron ions. The prepared cellulose-based conductive macrofiber has a dense fiber structure and possesses a tensile strength of 449 MPa (enable to lift 2 kg weights). Its electrical conductivity reaches as high as 5.32 S cm^−1^ due to the uniform distribution of conductive CNTs and PPy in the surrounding of the nanofiber. Degradation experiment demonstrates that BC/CNT/PPy conductive macrofiber can be completely degraded within 108 h, and the left conductive materials of CNTs and PPy can be collected and reused. In addition, the macrofiber can keep the dense fiber structure after soaking in water for 1 day, with tensile strength and conductivity just decreased by 6.7% and 8.1%, respectively. The developed fabric-based TENG utilizing BC/CNT/PPy conductive fiber as electrode shows a maximum open-circuit voltage of 170 V, short-circuit current of 0.8 µA, and output power at 352 μW, which is capable of powering the commercial electronics. Furthermore, the fabric-based TENGs attached to the human body can work as self-powered sensors to effectively monitor various motions (such as walking, running, jumping, arm lifting, arm bending, and leg lifting). This study suggests the potential of biodegradable, super-strong, and washable conductive cellulose macrofiber for designing fabric-based TENG for energy harvesting and biomechanical monitoring.

## Experimental Section

### Materials

The BC wet membranes were purchased from Hainan Yide Foods Co. Ltd. (China). The short multi-walled CNTs (MWCNTs, purity ≥ 98wt%, length = 0.5–2 μm) were purchased from Chengdu Organic Chemicals Co. Ltd. Cellulase was purchased from Beijing Soleibao Technology Co., Ltd. Cetyltrimethylammonium bromide (CTAB), pyrrole, sodium acetate, acetic acid, p-toluenesulfonic acid (P-TSA), chloroform, n-butanol, and 3,5-dinitrosalicylic acid are all purchased from Shanghai Biochemical Technology Co., Ltd. and used without further purification. Anthrone, concentrated sulfuric acid, potassium sodium tartrate, sodium bisulfite, recrystallized phenol, sodium hydroxide, and ferric chloride hexahydrate (FeCl_3_·6H_2_O) were all purchased from Shanghai Sinopharm Chemical Reagent Co., Ltd. and used without further purification.

### Preparation of BC Macrofibers

The BC wet membranes were firstly cut into strips with length of 15 cm and width of 7 mm and prepared for swelling in the boiling water. Then, the swollen striped BC hydrogels were wet-stretched by 30% at a speed of 2 mm min^−1^ under the mechanical force. Finally, the wet-stretched BC strips were twisted into macrofibers and then dried at room temperature for 12 h under tension to prepare the pure BC macrofibers.

### Preparation of BC/CNT Macrofibers

CNTs were adsorbed and embedded uniformly in BC using an aqueous CNTs dispersion containing a surfactant. Firstly, the CNTs were sonicated in aqueous solutions of CTAB, to obtain the CNTs dispersion solution. Then BC hydrogels were immersed in the CNTs dispersion solution with CNTs content at 0.025 wt% to 0.3 wt%, and 0.4 wt% CTAB, respectively. The solution was sonicated for 23 h and shaken at 150 rpm for 1 h. After repeating for 3 times, the CNTs could be well-dispersed in the BC to obtain the BC/CNT composite hydrogels. Finally, the BC/CNT composite macrofibers were prepared by the wet-stretching and wet-twisting. The composites obtained through different CNTs content of 0.025, 0.05, 0.1, 0.2, and 0.3 wt% were coded as BC/CNT-1, BC/CNT-2, BC/CNT-3, BC/CNT-4, and BC/CNT-5 macrofiber, respectively.

### Preparation of BC/CNT/PPy Macrofibers

After mechanical pressing to remove the physically adsorbed water, the striped BC/CNT-4 composite hydrogels were immersed in the iron (III) chloride hexahydrate under shaking. After the solution was shaken for 1 h, pyrrole (Py) solution with P-TSA was added and placed at 4 °C refrigerator for 1 h to initiate the polymerization. The molar ratio of FeCl_3_·6H_2_O: Py: P-TSA was 2.6:1:1 [53]. At the same time, the PPy was synthesized under the same conditions in the Fe^3+^ solution, used as a control sample for analysis experiment. After the reaction was completed, the obtained BC/CNT/PPy composite hydrogels were rinsed thoroughly with alcohol and distilled water in turn to remove excess reagents and other byproducts. Finally, the BC/CNT/PPy macrofibers were prepared through wet-stretching and wet-twisting the BC/CNT/PPy composite hydrogels. In this experiment, the Py solution with concentration of 0.01, 0.03, 0.05, 0.07, and 0.09 M was prepared to synthesize PPy in the BC/CNT-4 composite hydrogels, Corresponding BC/CNT/PPy macrofibers were coded as BC/CNT/PPy-1, BC/CNT/PPy-2, BC/CNT/PPy-3, BC/CNT/PPy-4, and BC/CNT/PPy-5 macrofiber, respectively.

### Degradation Experiment of BC/CNT/PPy Macrofibers

A HAc-NaAc buffer with a pH of 4.8 is prepared by mixing 150 mL of 0.2 M sodium acetate mother liquor with 100 mL of 0.2 M acetate mother liquor and finally diluting the volume to 1 L with distilled water. After dissolving 0.5 g of cellulase into 100 mL of HAc-NaAc buffer to obtain the enzyme solution, the BC macrofibers and BC/CNT/PPy-3 macrofibers were immersed in the enzyme solution (50 °C in a water bath) for degradation experiments, respectively. 2 mL of enzymatic hydrolysis solution is taken for testing total sugar content every 12 h from the beginning of degradation experiment until macrofibers are completely degraded. During the degradation process, we took the photos of the morphology to compare their morphology. Additionally, in the process of degradation, the available macrofibers were obtained for observing their morphology with SEM and calculating their weight loss. Finally, the total sugar concentration of the enzymatic hydrolysate was measured.

### Preparation of Fabric-Based TENG

Firstly, the BC/CNT/PPy macrofibers are woven into a nylon (thickness, ~ 3 mm) fabric to obtain the cellulose-based/nylon macrofiber fabric through “Inlay” technique, which can properly integrate the fiber in a straight-line configuration. Here, nylon is a common positive triboelectric material for the preparation of flexible wearable TENG [54, 55]. Then the conductive sliver thin membrane (thickness, ~ 0.1 µm) is pasted on the PDMS thin membrane (thickness, ~ 0.1 mm) and obtains PDMS/sliver film. Finally, the fabric-based TENG (10.5 cm by 6 cm) is constructed by using cellulose-based/nylon macrofiber fabric as one of the friction layers/electrodes and using PDMS/sliver film as the other friction layer/electrode.

### Characterization

The morphologies of the non-stretched and stretched BC, BC/CNT, BC/CNT/PPy hydrogels, and BC, BC/CNT-4, BC/CNT/PPy-3 macrofibers were observed utilizing S-4800 field emission scanning electron microscope. The mechanical tensile properties were evaluated using a Universal Testing Machine HOUNSFIELD, equipped with a 5000 N load cell with a crosshead speed of 5 mm min^−1^. The Fourier transform infrared spectra (FTIR), X-ray diffraction (XRD), elements analysis (EA), and X-ray photoelectron spectroscopy (XPS) were used to investigate the chemical compositions of the samples. The electrical conductivity of the composite macrofibers was tested using an electrochemical workstation. The optical density (OD) of enzyme hydrolysate was measured using a Spectrophotometer. The output performance of fabric-based TENG was evaluated assisted with a cyclic contact-separation process through a linear motor, and corresponding output was recorded by an electrostatic meter (Keithley 6514).

## Results and Discussions

### Morphology of BC/CNT/PPy Macrofibers

**Fig. 1:**
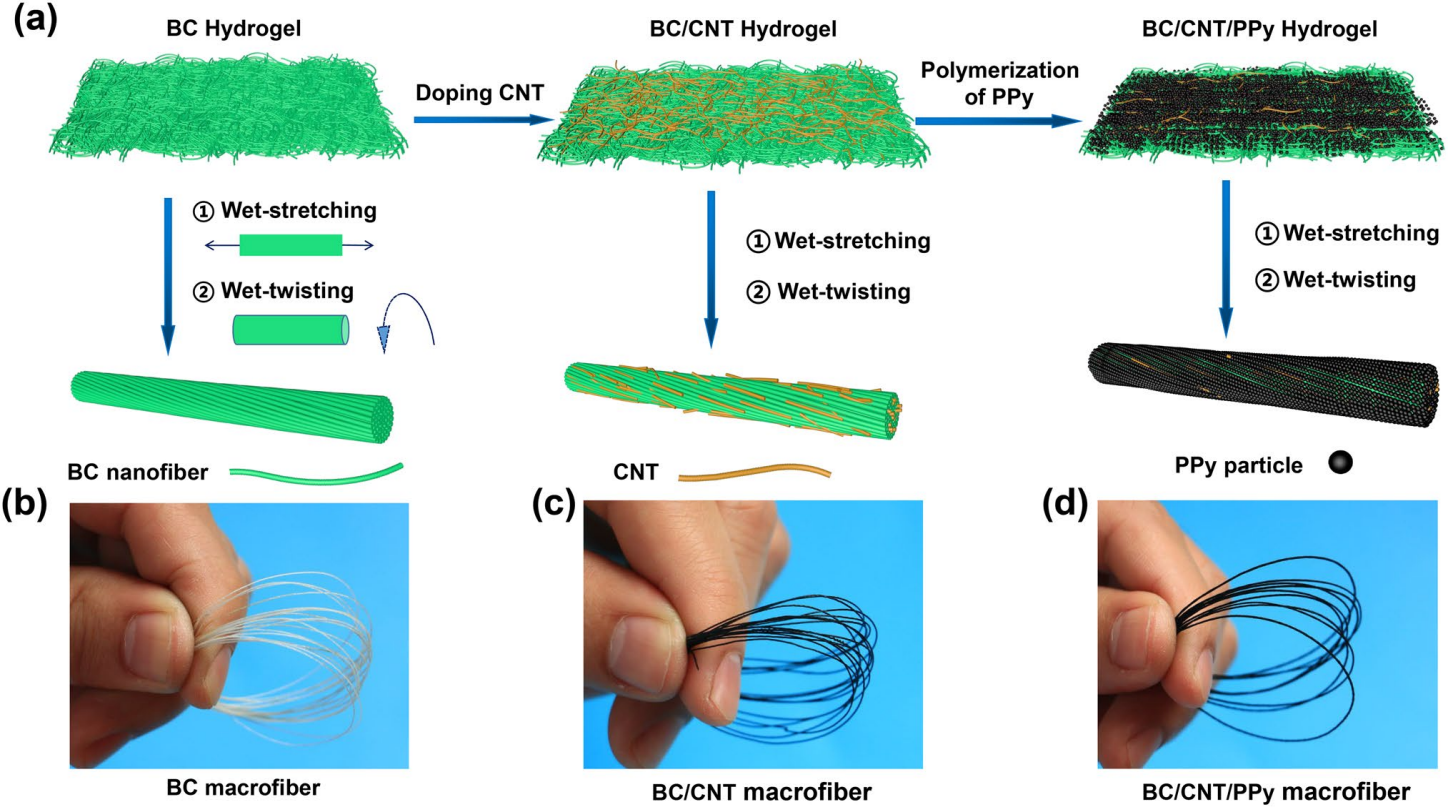
**a** Schematic illustration of BC, BC/CNT, BC/CNT/PPy macrofibers fabrication. The photographs of **b** BC, **c** BC/CNT, and **d** BC/CNT/PPy macrofibers

Figure 1a shows the schematic preparation process of pure BC, BC/CNT, and BC/CNT/PPy macrofibers. The preparation of corresponding hydrogel is schematically illustrated in Fig. S1. When the striped BC hydrogels are immersed in the CNTs dispersion solution, the CNTs (as nanoscale fillers) can be well-incorporated into BC hydrogels in the aqueous state with repeated shaking and sonication to obtain the striped BC/CNT hydrogels. Thereafter, BC/CNT hydrogels are immersed in the iron (III) solution after mechanical pressing to remove the physically adsorbed water. Thus, Fe^3+^ solution (as oxidant) is easier to cover on the BC/CNT hydrogels. After adding BC/CNT hydrogels (impregnated with Fe^3+^) into the aqueous pyrrole and *p*-toluenesulfonic acid (P-TSA) solution, PPy is formed on the surface of BC nanofibers through in-situ oxidative polymerization to obtain the striped BC/CNT/PPy hydrogel samples. Finally, the BC, BC/CNT, and BC/CNT/PPy macrofibers are obtained via wet-stretching and wet-twisting method. More detailed fabrication process is explained in the Experimental section. Here, for discussing the influence of CNTs and PPy content on the mechanical properties and electrical conductivity of the macrofibers, five types of BC/CNT macrofibers are prepared in the dispersion solution with different CNTs contents, and coded as BC/CNT-1 to BC/CNT-5 macrofiber, respectively; five types of BC/CNT/PPy macrofibers are prepared in the solution with different pyrrole concentrations, and coded as BC/CNT/PPy-1 to BC/CNT/PPy-5 macrofiber, respectively. The prepared macrofibers are shown in Fig. 1b–d. The pure BC macrofibers present white color, and the BC/CNT and BC/CNT/PPy macrofibers turn to black color due to incorporated with CNTs and coating with PPy. It should be noted that the BC/CNT-4 and BC/CNT/PPy-3 macrofibers show the best comprehensive performance about conductivity and mechanical properties in their own type of macrofibers from later characterization. Therefore, for the convenience of expression and unless otherwise specified, BC/CNT and BC/CNT/PPY macrofibers represent BC/CNT-4 and BC/CNT/PPy-3 macrofibers, respectively.

Figure S2 shows the SEM images of non-stretched and 30% stretched BC, BC/CNT, and BC/CNT/PPy hydrogels. As is shown in Fig. S2a, pure BC hydrogels have a 3D network structure composed of nanofiber, which is conducive to the doping of conductive nanomaterials. Therefore, CNTs with short tubular fiber structure (as conductive nanoscale fillers, Fig. S3a-b), have been well-dispersed on BC hydrogels via repeated operation of shaking and sonication (Fig. S2b). In addition, Fig. S2c shows the PPy particles (or small aggregates) precipitated via in-situ chemical polymerization evenly distributed in the network structure of BC. Obviously, the homogeneous dispersion and precipitation of CNTs and PPy can endow the composite higher electrical conductivity. Figure S3c-d shows the SEM and TEM images of PPy particles, which have larger particle size and more regular spherical structure compared with PPy synthesized inside the BC/CNT hydrogels. This is because the pores inside BC/CNT hydrogels are too small, which not only limit the synthesis of larger PPy particles, but also prevent Fe^3+^ from contacting with more pyrrole monomers. From the images of Fig. S2a-c, we can see that BC/CNT hydrogels have the densest structure among three types of hydrogels. This is because the CNTs fill in the pores of BC hydrogels, but then the network structure is expanded after the synthesis of PPy inside the BC/CNT hydrogels. As is shown in Fig. S2d-f, the SEM images show all type of hydrogels get better nanofiber alignment after 30% wet-drawing, which promises BC hydrogels more excellent mechanical properties [56, 57].

**Fig. 2:**
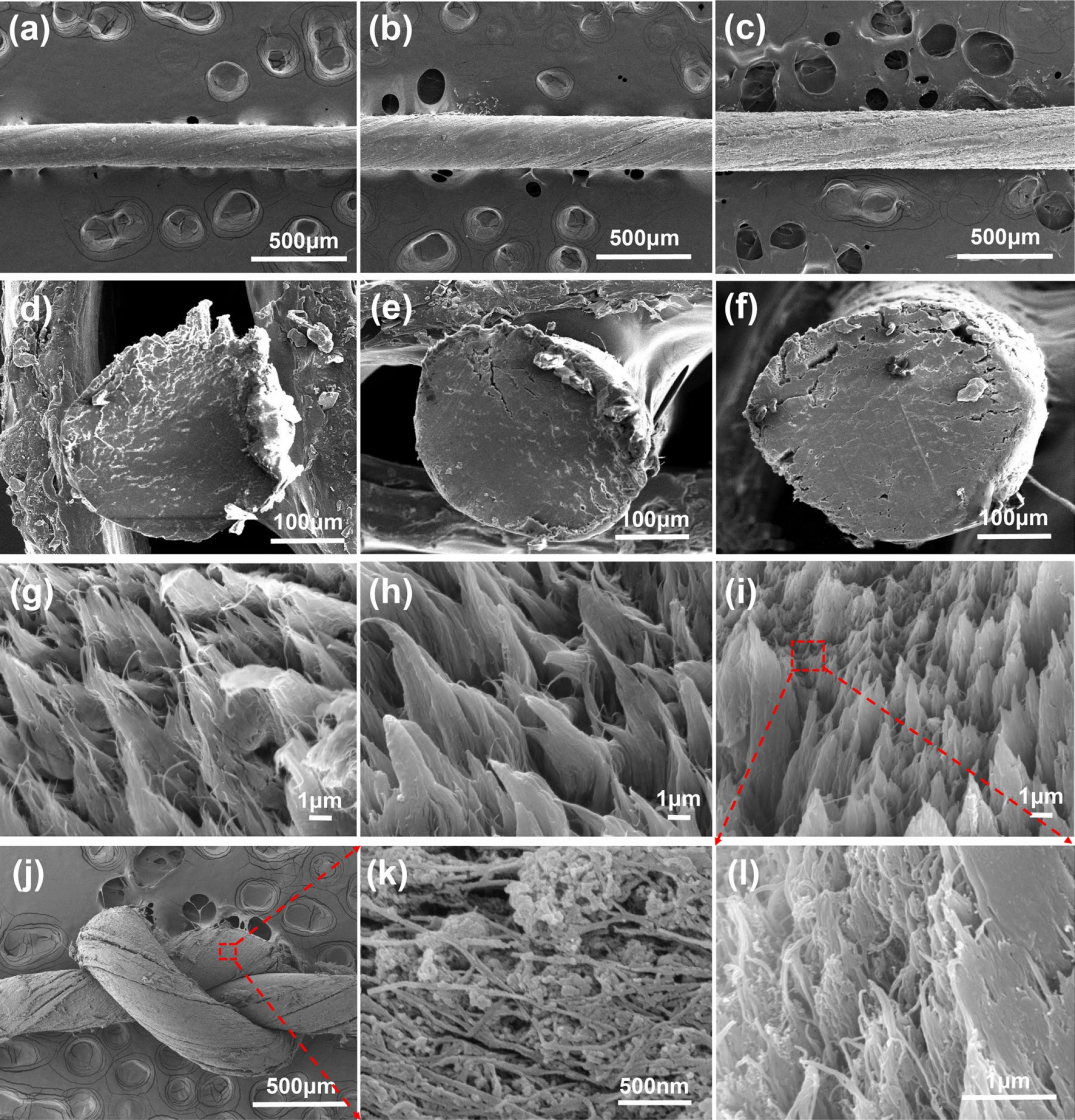
SEM images of pure BC, BC/CNT, BC/CNT/PPy macrofibers. Surface morphology of **a** BC, **b** BC/CNT, **c** BC/CNT/PPy macrofiber. Fracture surfaces of **d, g** BC macrofiber, **e, h** BC/CNT macrofibers, **f, i, l** BC/CNT/PPy macrofibers. **j** Knotted BC/CNT/PPy macrofiber and **k** the surface

Figure 2a–c shows the SEM images of the surface morphology of BC, BC/CNT, and BC/CNT/PPy macrofibers. With the incorporation of CNTs and in-situ synthesis of PPy, both the roughness of macrofibers surface and fiber diameter gradually increase. The SEM images of their fracture surface shows all types of macrofibers possess dense nanofiber structure after wet-twisting and drying under a tension force (Fig. 2d–i), which is capable of improving mechanical properties of the macrofibers. From the enlarged image of Fig. 2k, we can see that the PPy particles uniformly distributed on the surface of BC/CNT/PPy macrofibers. In addition, PPy particles with smaller size also exist inside the BC/CNT/PPy macrofibers (Fig. 2l). It is obvious that the uniform distribution of PPy can endow the macrofibers excellent electrical conductivity. More importantly, the BC/CNT/PPy macrofibers can be knitted without breaking (Fig. 2j), which are ready to be woven into fabrics. The polymerization process of PPy is summarized in Fig. S4. To confirm the successful substitution of P-TSA in the benzene ring, the mappings of C, N, O, and S images for PPy particles are obtained. In Fig. S5, the O and S atoms evenly distribute in the framework of carbon atoms, showing the successful P-TSA grafting onto PPy. Additionally, the mappings of C, N, O, and S images for the fracture surface of BC/CNT/PPy macrofibers demonstrate that the PPy particles doped with P-TSA are uniformly distributed inside macrofibers (Fig. S6).

### Characterization of BC/CNT/PPy Macrofibers

**Fig. 3:**
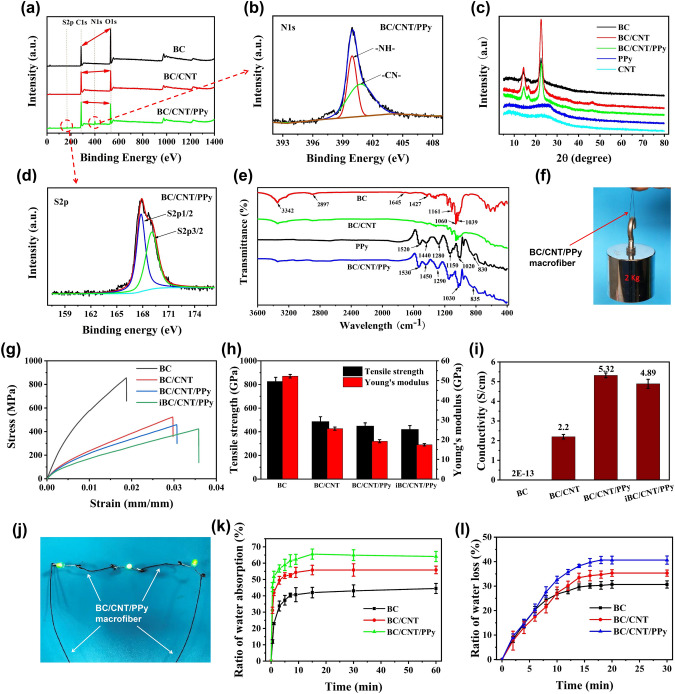
**a** Wide scans measured for BC, BC/CNT, BC/CNT/PPy macrofibers, PPy and corresponding **b** N 1* s* and **d** S 2*p* core-level spectra of PPy and BC/CNT/PPy macrofiber. **c** XRD patterns of BC, BC/CNT, BC/CNT/PPy macrofiber, PPy, and CNT. **e** FTIR spectra of BC, BC/CNT, BC/CNT/PPy macrofibers, and PPy. **f** BC/CNT/PPy macrofiber lift 2 kg weight. **g** Stress–strain curves, and **h** tensile strength and Young’s modulus of BC, BC/CNT, BC/CNT/PPy, and iBC/CNT/PPy macrofibers. **i** The electrical conductivity of BC, BC/CNT, BC/CNT/PPy, and iBC/CNT/PPy macrofibers. **j** BC/CNT/PPy macrofibers as a conductive yarn to light up LED when the power is turned on. The ratio of water absorption **k** and water loss **l** of BC, BC/CNT and BC/CNT/PPy macrofibers

X-ray photoelectron spectroscopy (XPS) conducted on the BC composite macrofibers indicates that the systematic reaction has been successfully achieved. The comparative wide scans displayed in Fig. 3a show that all macrofibers have binding energies (BE) consistent with carbon 1* s* (C 1* s*) and oxygen 1* s* (O 1* s*) core-shells, with a significantly increased content in carbon after CNTs and PPy loading along with an expected escalating difference in C 1* s*/O 1* s* (duly marked in the spectra). In BC/CNT/PPy macrofibers, the N 1* s* core-level peak can be fit into two peak components at 399.3 and 400.4 eV, as ascribed to N–H and N–C in the ring of pyrrole, respectively; S 2*p* can be deconvoluted into two peaks, which can be assigned to the -SO_3_ group of PTS at 167.5 and 169.5 eV, and belongs to the binding energy of S 2*p*_1/2_ and S 2*p*_3/2_, respectively (Fig. 3b, d). The results further prove the successfully synthesis of PPy doped with P-TSA in the BC/CNT/PPy macrofibers. The XRD patterns of BC composite macrofibers, CNTs, and PPy particles are displayed in Fig. 3c. The BC macrofiber exhibits characteristic peaks at 2θ = 14.6°, 16.8°, and 22.8°, which are indexed as (110), (101), and (020) for cellulose I [58]. Additionally, the XRD results show that the characteristic peaks of CNTs and PPy are broad in reflection peaks, which are typical characteristics of the amorphous state.

Figure 3e shows the Fourier transform infrared (FTIR) spectra of BC macrofibers and PPy particles. The peaks of –OH stretching vibration and C-H asymmetrically stretching vibration of BC are found in the region of 3342 and 2897 cm^−1^, respectively. The band at 1645 cm^−1^ represents O–H bending of absorbed water. The bands at 1060 and 1161 cm^−1^ are the peaks of C–O and C–O–C, respectively, as confirmed in previous work [59]. For the spectra of the PPy and BC/CNT/PPy macrofibers, the peaks shift to higher wavelength values. For instance, in the BC/CNT/PPy macrofibers, the peaks of 1530 cm^−1^ is ascribed to C=C in the aromatic ring of pyrrole. The peaks at 1450, 1290, 1030, and 835 cm^−1^ correspond to C–C, C–N stretching aromatic amine, =C–H bending, and N–H wagging out of plane of PPy ring, respectively. The blue-shift of these bands confirms the presence of the chemical interactions between the -H of the secondary amine in the pyrrole ring or the -H in the benzene ring of P-TSA and the surface -OH groups in BC.

The typical stress–strain curves and corresponding tensile strength, Young’s modulus for BC, BC/CNT, BC/CNT/PPy, and iBC/CNT/PPy macrofibers (iBC/CNT/PPy sample is denoted as the BC/CNT/PPy macrofiber immersed in water for 1 day and then dried) are depicted in Fig. 3g–h. From the image of Fig. 3g, we can see that the pure BC macrofibers have the highest mechanical strength compared with other composite macrofibers. Corresponding tensile strength and Young’s modulus are 825.7 MPa and 52.2 GPa (Fig. 3h), which are the same level value as described in previous work [60]. This high stiffness is related to the unique properties of BC where highly oriented nanofibers are tightly intertwined together after wet-stretching and wet-twisting. After incorporation of CNTs and polymerization of PPy, due to the looser structure of cellulose nanofibers, the tensile strength and Young’s modulus of BC/CNT/PPy macrofibers decrease to 449 MPa and 19.6 GPa, which is still superior to previous conductive macrofibers (Fig. S7), such as CNT-cotton [21], PEDOT:PSS-CNT [61], PI/Cu/PET [62], GO-CMC [63], GO [64], etc. As is shown in Fig. 3f, the macrofiber can lift 2 kg weight, demonstrating the developed BC/CNT/PPy macrofibers possess excellent mechanical strength and are conducive to fabricating fabric-based TENG with excellent stability and durability. More importantly, after immersing in the water for 1 day and dried at room environment, the iBC/CNT/PPy macrofibers still hold the 418.6 MPa of tensile strength and 17.9 GPa of Young’s modulus (only decrease by 6.6% and 8.7%), exhibiting good structural stability of the macrofibers in water.

To further investigate the influence of CNTs and PPy contents on the mechanical properties of BC macrofibers, the BC/CNT macrofibers with different CNTs contents and the BC/CNT/PPy macrofibers with different PPy contents have been prepared. Firstly, the element content of all BC composite macrofibers is measured by element analysis as shown in Tables S3 and S4. For macrofibers of the BC/CNT group, the calculated content of CNTs gradually increases, and corresponding tensile strength and Young's modulus show a trend of gradually decrease (from 680.8 MPa and 45 GPa of BC/CNT-1 macrofiber with 2.36 wt% CNTs content to 376.3 MPa and 12.5 GPa of BC/CNT-5 macrofiber with 8.5 wt% CNT content, respectively). This is because higher content of carbon nanotubes causes looser nanofiber structure of BC. The results demonstrate that the mechanical strength of BC/CNT macrofibers is dependent on the content of CNT. For BC/CNT/PPy macrofibers, with the increase of pyrrole concentration, the content of N and S elements experience a change of gradual increase and then decease, which is an evidence of the variation PPy content. The maximal value (30% PPy content) is observed in the sample of BC/CNT/PPy-4. The subsequent decrease of PPy content may be the formation of PPy aggregate blocks due to high concentration of pyrrole monomer, which blocks the BC network and prevents further polymerization of PPy. As is shown in Table S4, the tensile strength and Young’s modulus of BC/CNT/PPy macrofibers firstly display an increase (from 450.4 MPa and 23.4 GPa of BC/CNT/PPy-1 macrofiber to 515.9 MPa and 29.3 GPa of BC/CNT/PPy-2 macrofiber). Then they gradually decrease to 317.1 MPa and 13.8 GPa of BC/CNT/PPy-3 macrofiber, and finally sharply decrease to 136.3 MPa and 5.1 GPa of BC/CNT/PPy-5 macrofiber. The above results illustrate that appropriate polymerization of PPy can improve the mechanical properties of BC/CNT macrofibers, while the excessive polymerization of PPy can deteriorate the dense structure and reduce the mechanical strength of macrofibers.

The electrical conductivity of electrodes is crucial to improve the output performance of TENG, because the higher electrical conductivity is conducive to the charge transfer during contact electrification. The electrical conductivity of BC composite macrofibers is depicted in Fig. 3i, which shows that the incorporation of CNTs and polymerization of PPy can effectively improve the conductivity of cellulose macrofibers. As is shown in Fig. 3j, the BC/CNT/PPy macrofibers with conductivity of 5.32 S cm^−1^ can be readily used as cables for lighting up LEDs. Additionally, the iBC/CNT/PPy macrofibers can maintain a conductivity of 4.89 S cm^−1^ after immersing in water (just loss 8.1%), the resistance is ~ 718 Ω. As is shown in Table S3, the conductivity of BC/CNT macrofibers firstly increases with the increased CNTs contents (reaching the highest value of 2.22 S cm^−1^ for BC/CNT-4 macrofiber with 7.14% CNTs content) and then decreases to 1.72 S cm^−1^ for BC/CNT-5 macrofiber with 8.5% CNTs content (probably induced by the agglomeration of CNTs). Therefore, owing to the highest conductivity of BC/CNT-4 macrofibers, the BC/CNT-4 hydrogels are selected for further in-situ polymerization of PPy to prepare BC/CNT/PPy macrofibers. As is shown in Table S4, the conductivity of BC/CNT/PPy macrofibers gradually increases to a stable value with PPy content in BC/CNT/PPy macrofiber at 23.14%. We can see that the conductivity of the BC/CNT/PPy macrofiber containing 26.74% PPy is slightly higher than the macrofiber containing 30% PPy. This may be because the larger the concentration of pyrrole monomer, the larger the diameter of the synthesized PPy nanoparticles, which may lead to serious aggregation phenomenon between each other. Severe aggregation will block the fiber network of BC, hindering the synthesis of PPy inside BC. Therefore, the connectivity of PPy nanoparticles to each other will become poor, resulting in reduced electrical conductivity. Hence, BC/CNT/PPy-3 macrofibers are selected as standard materials for subsequent characterization and applications. According to the above results, CNTs and PPy are promising to be used as conductive nanofiller and can effectively improve the conductivity of BC macrofibers.

The weight change of BC macrofibers immersed in water is also explored. Figure 3k shows the ratio of water absorption of BC, BC/CNT, and BC/CNT/PPy macrofibers, indicating that almost all macrofiber samples reach the equilibrium state of water absorption after 15 min. Additionally, the ultimate ratio of water absorption of BC, BC/CNT, and BC/CNT/PPy macrofibers is approximately 40%, 55%, and 65%, respectively. It can be explained that the macrofibers have looser internal structure after the incorporation of CNTs and polymerization of PPy, which can retain more water compared with pure BC macrofiber. Furthermore, the ratio of water loss for the macrofibers dried at room temperature has been studied. The results show all macrofibers can be completely dried within 20 min (Fig. 3l). According to the above results, the excellent mechanical properties and electrical conductivity of BC/CNT/PPy macrofibers upon water immersion tests illustrate the developed conductive cellulose-based macrofibers are promising materials for preparing washable functional fabrics. Additionally, the air permeability of cellulose-based/nylon macrofiber fabric was performed (Fig. S8), which improves with the increased pressure. The woven fabric shows highest air permeability of 2,778 mm s^−1^, which is still comparable to most ordinary clothing fabrics. These results demonstrate that the cellulose/nylon macrofiber fabric with knitting structure has outstanding breathability.

### Degradability of BC/CNT/PPy Macrofibers

**Fig. 4:**
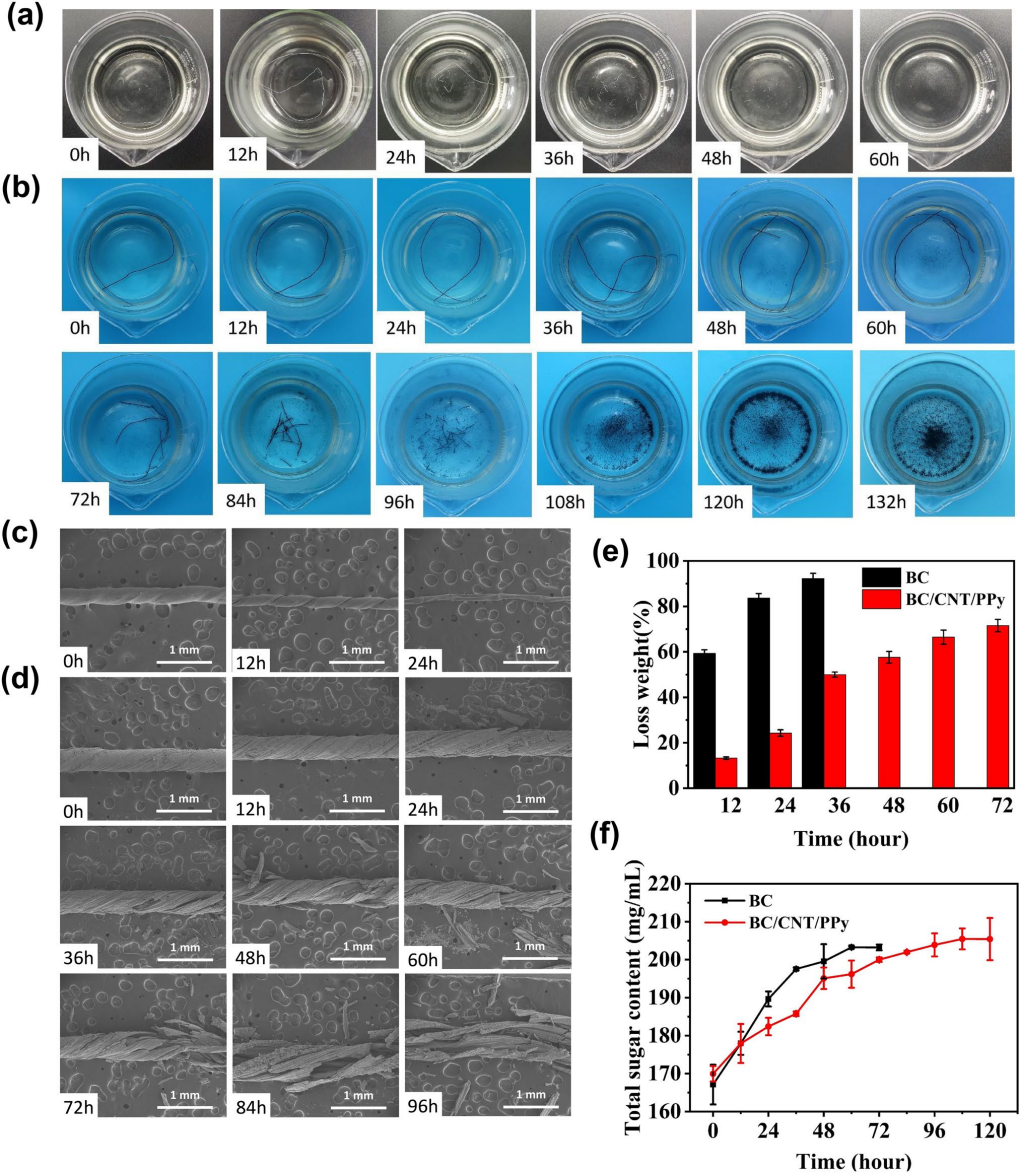
The degradation experiment of BC and BC/CNT/PPy macrofiber in the cellulase solution. The photographs of macrofibers during degradation, **a** BC macrofiber, **b** BC/CNT/PPy macrofiber. SEM images of macrofibers, **c** BC macrofiber, and **d** BC/CNT/PPy macrofiber. **e** The loss weight of BC and BC/CNT/PPy macrofibers during degradation. **f** Total sugar content in the cellulase solution of BC and BC/CNT/PPy macrofibers during degradation

Previous literatures have reported the biodegradability of BC hydrogels in the cellulase solution [40], which is capable of degrading cellulose into glucose owing to the decomposition of *β*-1, 4 glycosidic bonds. However, it is unclear whether the conductive cellulose-based macrofibers with dense nanofiber structure via wet-stretching and wet-twisting method can be completely degraded. Hence, we investigate and evaluate the degradation experiments of BC and BC/CNT/PPy macrofibers with enzymatic degradation method. Figure 4a–b presents the photo images of real-time degradation process every 12 h, and we can see that the BC and BC/CNT/PPy macrofibers gradually degrade in cellulase solution. The SEM images of BC macrofibers during 24h degradation process (Fig. 4c) indicate that the diameter of macrofiber gradually decreases, during which it is also companied with simultaneously fragmentizing until complete degradation. For the BC/CNT/PPy macrofibers, due to the coating of PPy on the nanofiber, the degradation process is slower than BC macrofiber. After the BC nanofibers inside the macrofiber are degraded, the PPy aggregates start to disrupt in the cellulase solution, which cause the macrofibers to be further broken (Fig. 4d). Finally, the BC/CNT/PPy macrofibers are completely degraded, leaving only conductive CNTs and PPy. In addition, the weight change of macrofibers is recorded during degradation, as is shown in Fig. 4e. Obviously, the degradation rate of BC macrofiber is faster than BC/CNT/PPy macrofiber. Specifically, during the degradation process, the mass of BC macrofiber is reduced by ~ 60% within 12 h, and by ~ 90% within 36 h; the mass of BC/CNT/PPy macrofiber is reduced by ~ 50% within 36 h, and by ~ 70% within 72 h. It is reflected that the degradation of BC macrofiber mainly occurs in the first 36 h, while the degradation of BC/CNT/PPy macrofiber is faster in the first 36 h and tends to be slower in later time. Meanwhile, the total sugar content in the cellulase solution is measured during the degradation process (Fig. 4f). It further proves that the degradation of BC macrofiber is faster than BC/CNT/PPy macrofiber. In addition, after 60 and 108 h, the total sugar content in the cellulase solution of BC and BC/CNT/PPy macrofibers does not change significantly. It is suggested that BC and BC/CNT/PPy macrofibers can be completely degraded within 60 and 108 h, respectively. Therefore, biodegradable BC/CNT/PPy macrofiber as the electrode and triboelectric materials is significant and has great potential for preparing fully degradable fabric-based TENG.

### Performance Characterization of Fabric-Based TENG

**Fig. 5:**
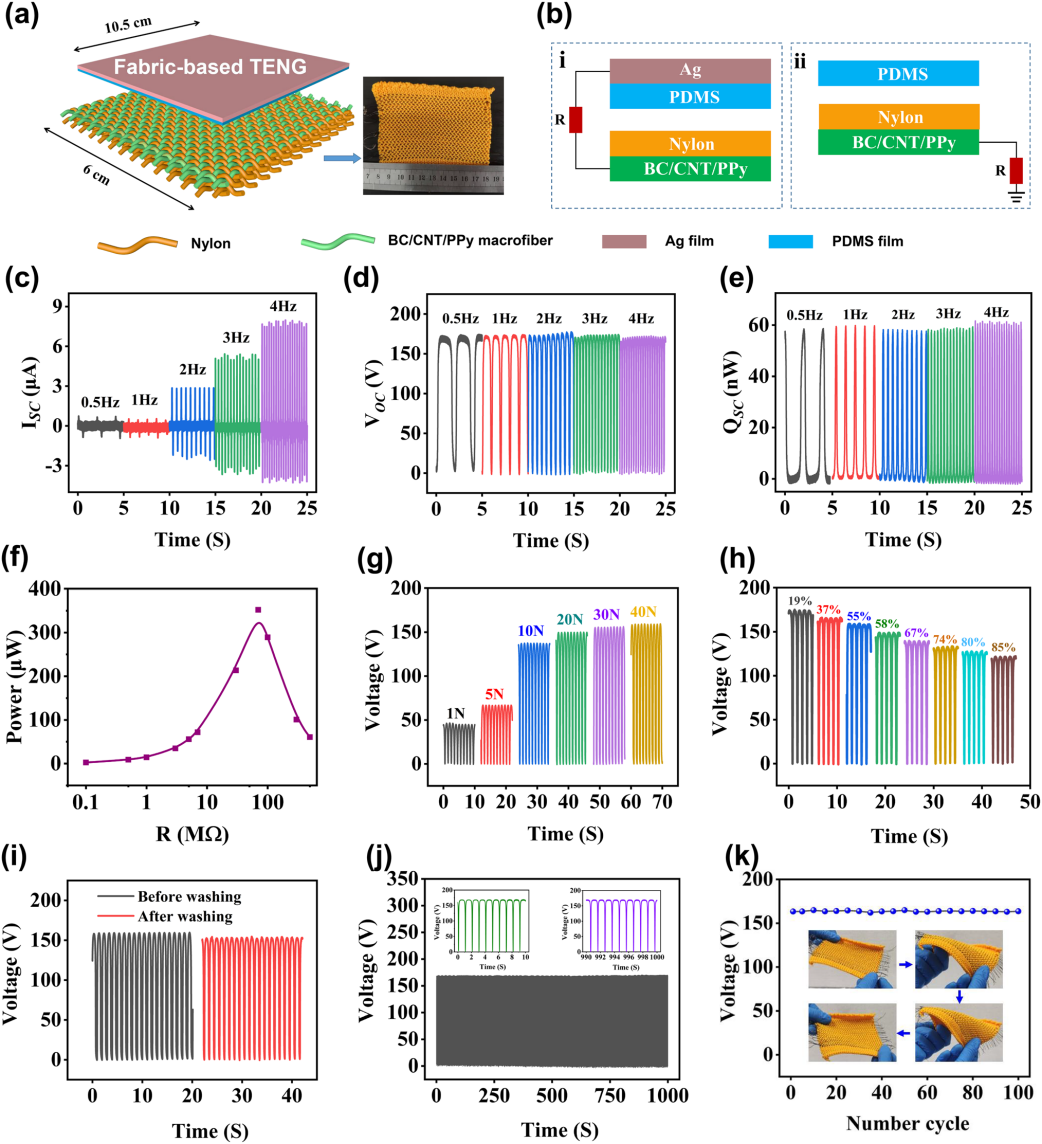
**a** Schematic diagram of fabric-based TENG structure; **b** two working modes of fabric-based TENG, (i) contact-separation mode and (ii) single electrode mode. Electrical output performances and demonstration of the self-charging of the fabric-based TENG. **c** Short-circuit current, **d** open-circuit voltage and **e** transferred charge of the fabric-based TENG in various frequencies. **f** Instantaneous power as a function of external load resistance. **g** Output voltage of fabric-based TENG under different applied impact forces at 1 Hz. **h** Output voltage of fabric-based TENG with varying humidity. **i** Output voltage of fabric-based TENG before and after washing. **j** Output voltage of fabric-based TENG within 1000 s at contact-separation frequency of 1 Hz. **k** Output voltage of fabric-based TENG under 100 cycles of mechanical deformation

As is shown in Fig. 5a–b, the BC/CNT/PPy macrofiber is firstly woven into a nylon cloth through “Inlay” technique, which can properly integrate the fiber in a straight-line configuration. Then the fabric-based TENG is constructed by using BC/CNT/PPy macrofiber woven nylon cloth as one of the friction layers/electrodes and using PDMS/sliver film as the other friction layer/electrode. The designed fabric-based TENG can work in two different working modes: (i) contact-separation mode and (ii) single electrode mode (Fig. 5b). The contact-separation mode uses nylon cloth and PDMS as the triboelectric layers, and BC/CNT/PPy macrofiber and silver film as the electrodes for energy harvesting. For the single electrode mode, the BC/CNT/PPy fiber serves as the electrode, PDMS and nylon cloth serves as the other friction layer. This mode of TENG is used as a self-powered biomechanical sensor in this work. The working mechanism of the fabric-based TENG related with the coupling of contact electrification and electrostatic induction is schematically illustrated in Fig. S9. As is shown in Fig. S9a (contact-separation mode), as the electronegativity of PDMS and nylon is different, contact electrification will occur. The surface of nylon will be positively charged due to loss of electrons, while PDMS film will be negatively charged due to capture of electrons. In the process of separation, electrostatic induction induced by triboelectric potential will drive flowing of electrons between the Ag film and BC/CNT/PPy macrofiber electrodes of the TENG, yielding an output current through the external circuit. In the re-contact process, the reduction of the distance will make the top Ag electrode possess a higher electric potential than the bottom BC/CNT/PPy macrofiber electrode. As a consequence, electrons are driven from the bottom electrode back to the top electrode, inducing a reverse output current. As is shown in Fig. S9b (single electrode mode), the surface of PDMS and nylon cloth periodically contacts with each other under an external force. According to the triboelectric series, electrons transfer from nylon cloth to the PDMS since PDMS is more electronegative. Once there is a relative separation between PDMS and nylon cloth, electrostatic induction induced by triboelectric potential will drive flowing of electrons from ground to the BC/CNT/PPy macrofiber electrode, yielding an output current through the external circuit. When nylon cloth comes into contact with PDMS, the decreased triboelectric potential will drive free electrons to flow from the BC/CNT/PPy macrofiber electrode to ground and generate a reverse output current.

Many works on TENG for scavenging biomechanical energy are reported [65–67], whereas the advantage of fabric-based TENG is that it can be perfectly fitted to the body on a large scale to harvest the energy of human motion. The output performance of fabric-based TENG based on contact-separation mode is evaluated upon a linear contact-separation process. The effective contact area and contact distance of the two friction materials are 63 cm^2^ and 8 cm, respectively. The humidity of the test environment is 19%. The corresponding outputs are recorded by an electrostatic meter (Keithley 6514). The periodic contact and separation process will lead to an alternating output. As shown in Fig. 5c–e, when the contact-separation frequency increases from 0.5 to 4 Hz, the amplitude of the open-circuit voltage (*V*_OC_) and transferred charges (*Q*_SC_) keep almost unchanged at 170 V and 60 nC, while the amplitude of the short-circuit current (*I*_SC_) increases from 0.7 to 7.5 µA. According to the effective contact area of fabric-based TENG, the calculated maximum current density and charge density under contact-separation in 4 Hz are 0.115 µA cm^−2^ and 0.92 nC cm^−2^ respectively. The relationship between output voltage of fabric-based TENG and frequency can be explained as follows. According to the Gauss theorem [68–70], for the contact-separation mode of TENG, the *V*_OC_ is given by:

1 $$Voc = - \frac{Q}{{S\varepsilon_{0} }}\left( {\frac{{d_{1} }}{{\varepsilon_{1} }} + \frac{{d_{2} }}{{\varepsilon_{2} }} + x\left( t \right)} \right) + \frac{\sigma x\left( t \right)}{{\varepsilon_{0} }}$$

where *Q* is the amount of transferred charges and ${ }x$ is the separation distance between the two triboelectric charged layers, *ε*_0_, *S*, and *σ* are the permittivity of the vacuum, the electrode area and static charges density on the triboelectric layer, respectively. *d*_1_ and *d*_2_ are the thickness, and *ε*_1_ and *ε*_2_ are the relative dielectric constants of two triboelectric layer. Equation (1) is the basic equation for the contact-separation mode of TENG and can be utilized to calculate its output property. First, two special cases of the open-circuit (OC) condition and short-circuit (SC) condition are analyzed. At OC condition, there is no charge transfer, which means that Q is 0. Therefore, the open-circuit voltage *V*_OC_ is given by:

2 $$Voc = \frac{\sigma x\left( t \right)}{{\varepsilon_{0} }}$$

We can see that the *V*_OC_ (as a function of both $x\left( t \right)$ and the static charge density of *σ*) is mainly determined by the structures and the selected materials of the fabric-based TENG, which shows no response to frequency. For the single electrode mode, where d_2_ = 0, the result is the same.

At SC condition, *V* is 0. The effective thickness constant d_0_ was defined as d_1_/ε_1_ + d_2_/ε_2_. Therefore, the transferred charges are:

3 $$Qsc = \frac{S\sigma x\left( t \right)}{{d_{0} + x\left( t \right)}}$$

From Eq. (3), the short-circuit current is:

4 $$Isc = \frac{dQsc}{{dt}} = \frac{{S\sigma d_{0} }}{{\left[ {d_{0} + x\left( t \right)} \right]^{2} }}\frac{dx}{{dt}} = \frac{{S\sigma d_{0} v\left( t \right)}}{{\left[ {d_{0} + x\left( t \right)} \right]^{2} }}$$

It can be seen from the above equation that the current is related to the speed of contact-separation, that is, it varies with the frequency. Additionally, the surface charge density increases with the number of contacts between the two dielectric materials and eventually reaches saturation; hence, the charge doesn't change with frequency (Fig. 5e).

Usually, the effective output power of the TENG depends on the external loading resistance. Figure S10 shows the resistance dependent output voltage and output current with load resistance ranging from 0.1 to 500 MΩ. The output voltage of the TENG rises up with increased loading resistance, while the current density drops with the increased resistance due to the Ohmic law. As is shown in Fig. 5f, the output power (*W* = *I*_peak_^2^ $\times$ *R*) is plotted as a function of the loading resistance. It firstly shows a rising trend until the maximum output power reaches 352 µW (maximum power density is 54.14 mW m^−2^) at a loading resistance of 70 MΩ, and then it shows a drop trend. The output power density is comparable to other fabric-based TENG composed of metal materials as electrodes, such as stainless-steel wire (60 mW m^−2^) [71], Ag-plated fiber (11 mW m^−2^) [13], Cu-coated PET fiber (15.5 mW m^−2^) [62], and carbon black fiber (130 mW m^−2^) [72]. The real-time *V*_OC_ sensing signals according to the applied different force are also recorded in Fig. 5g, which steadily increase from 46 to 160 V with the force increased from 1 to 40 N. The increased *V*_OC_ can be attributed to that the applied increased force leads to the fabric-based TENG expansion with an enlarged deformation magnitude, which increases the contact area between friction materials. The output behavior of fabric-based TENG with varied relative humidity was performed. As is shown in Fig. 5h, the voltage signal of the fabric-based TENG gradually decreases from 167 to 123 V with the humidity increased from 19 to 85%. The decrement trend can be assigned to several reasons induced by increased humidity, including poorer charge accumulation, improved contact resistance, reduced electron transfer process, etc. Washability is an important index to evaluate the output stability of fabric-based TENG, Fig. 5i shows the *V*_OC_ output of fabric-based TENG before and after washing, there is small variation in the electrical output after washing. The result implies the sufficient washability of prepared fabric-based TENG for daily usage. As is shown in Fig. 5j, no obvious decay was observed during the 1000 compressing-releasing cycles and the 100 cycles of large-range twisting and stretching (Fig. 5k), showing excellent stability for practical applications.

### Fabric-Based TENG for Powering Electronic Devices

**Fig. 6:**
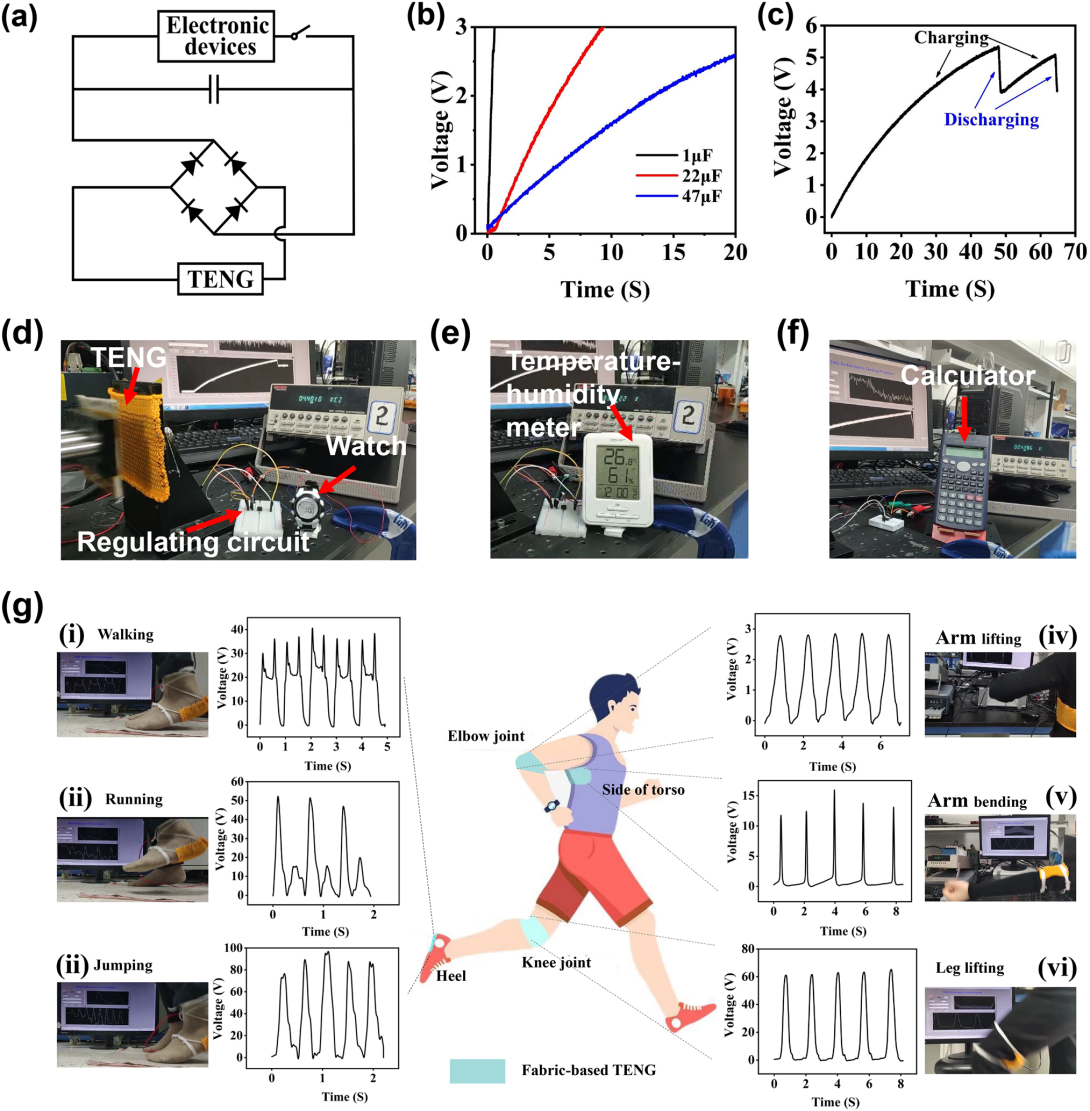
The developed applications of fabric-based TENG. **a** Diagram of fabric-based TENG charging capacitors and powering electronics. **b** Charging curve of commercial capacitors by pressing the fabric-based TENG. **c** Real-time test of the capacitor voltage and powering a watch. **d** A watch, **e** a temperature–humidity meter, and **f** a calculator are driven by pressing the fabric-based TENG. **g** Testing photograph and output voltage signals of fabric-based TENG as self-powered sensor fixed to various parts of human body (heel, side of torso, elbow, and keen joint) to monitor mechanical motion, (i) walking, (ii) running, (iii) jumping, (iv) arm lifting, (v) arm bending, and (vi) leg lifting

The relatively high output power of the fabric-based TENG promises it to work as a power source for driving wearable portable electronics. Generally, the electricity generated from the fabric-based TENG can be stored in commercial capacitors by converting the output alternating current (AC) to direct current (DC) through the rectifier bridge circuit (Fig. 6a). Figure 6b and S11 exhibit the charging process of different capacitors (1, 22, 47, and 100 µF) under the contact-separation frequency of 4 Hz. This result shows that the fabric-based TENGs can be used to charge the capacitors, but the charging velocity is decreased with the increased capacitance. The capacitor can be charged to 53 V in 46 s and then power an electronic watch (Fig. 6c). Figure 6d and Movie S1 show the powering of the electronic watch (DFYJ.CO NT-62, 0.12 mW) by using fabric-based TENGs with a 22 µF capacitor. As is shown in Fig. 6e–f, the digital temperature–humidity meter (BLX346, 0.15 mW, 3.6 V) and calculator (SUNWOOD, EC-1882; 0.1 mW) are also successfully driven through the fabric-based TENG charged capacitors of 47 and 100 µF, respectively. Corresponding supporting videos are displayed in Movies S2 and S3. These results demonstrated that the prepared fabric-based TENG possesses a promising practical application in the areas of wearable electronics and self-powered systems.

### Fabric-Based TENG as a Self-Powered Sensor for Human Motion Monitoring

As the output of fabric-based TENG in single electrode mode is relatively lower but closely related with the mechanical displacement, it is more convenient to work as a self-powered sensor to monitor the motion of human body. For expanding the potential applications of fabric-based TENG in human motion monitoring, we monitored a series of activities of human (walking, running, jumping, arm lifting, arm bending, and leg lifting) by utilizing the fabric-based TENG as self-powered sensor and fixing to the heel, side of torso, elbow joint, and keen joint, as schematically illustrated in Fig. 6g. By fixing the fabric-based TENG to the heel of human, when the tester was walking, running, and jumping, the maximum output *V*_OC_ was ~ 40, ~ 50, and ~ 90 V, respectively (Fig. 6g(i–iii) and Movies S4-S6). We can see that the output *V*_OC_ increases gradually maybe due to the pressure force applied to the fabric-based TENG increases from the exercise form of walking, running to jumping. As is shown in Fig. 6g(iv–vi) and Movies S7-S9, other activities (arm lifting, arm bending, and leg lifting) also can be monitored by fixing the fabric-based TENG to the side of torso, elbow joint, and keen joint, which output *V*_OC_ was approximately 13, 2.7, and 60 V, respectively. From these results, the output of fabric-based TENG as wearable self-powered sensor for monitoring the various motions is obviously different, showing excellent sensing performance, which possess the potential applications in athlete training, fitness exercises, and physical rehabilitation of patients, etc.

## Conclusion

In summary, a cellulose-based, biodegradable, super-strong, and conductive macrofibers with a diameter 0.45 mm is developed by wet-stretching and wet-twisting the BC hydrogel incorporated with CNTs and PPy, which is successfully used to design wearable fabric-based TENG for energy harvesting and biomechanical motion monitoring. The cellulose-based macrofibers possess tensile strength of 449 MPa and electrical conductivity of 5.32 S cm^−1^, meanwhile capable of maintaining good structural stability in water. The degradation experiment demonstrates the macrofibers can be degraded within 108 h in the cellulase solution, exhibiting its good environmental friendliness. The designed fabric-based TENG with cellulose-based conductive macrofibers as electrodes shows a maximum open-circuit voltage of 170 V, short-circuit current of 0.8 µA, and output power at 352 μW, which can drive commercial electronics such as electronic watch, temperature–humidity meter, and calculator. Additionally, the prepared fabric-based TENG as self-powered sensors can effectively monitor the human movement of walking, running, jumping, arm lifting, arm bending and leg lifting. We expect that this developed fabric-based TENG composed of biodegradable cellulose macrofiber possess a promising application in many area of wearable electronics, self-powered systems, athlete training, and physical rehabilitation of patients.

## Supporting Information

The schematic illustration of preparation process of BC/CNT and BC/CNT/PPy hydrogels. SEM images of unstretched and stretched hydrogels of pure BC, BC/CNT, BC/CNT/PPy. SEM and TEM images of CNTs and PPy. Synthesis mechanism of PPy with P-TSA as a dopant. SEM of PPy particles and corresponding EDS elements. Fracture SEM of BC/CNT/PPy macrofibers and corresponding EDS elements. The charging curve of the 100 μF by pressing the fabric-based TENG at 4 Hz. The air permeability of woven cellulose-base/nylon macrofiber fabric. The energy-generating mechanism of the fabric-based TENG. Relationship between current and voltage of the external load. The charging curve of the 100 μF by pressing the fabric-based TENG at 4 Hz. The table of summary and comparison of fabric-based TENGs based various electrodes. The table of summary of degradable or green materials for fabricating TENG. The table of elementary analysis of BC/CNT macrofibers, CNT content, electrical conductivity, tensile strength and Young’s modulus. The table of elementary analysis of BC/CNT/PPy macrofibers, CNT content, electrical conductivity, tensile strength and Young’s modulus. The video of the fabric-based TENG powering a watch. The video of fabric-based TENG powering a temperature-humidity meter. The video of fabric-based TENG powers a calculator. The video of fabric-based TENG as self-powered sensor monitoring the walking of human, running, jumping, raising arm, bending arm and lifting leg of human.

## Supplementary Information

Below is the link to the electronic supplementary material.

Supplementary file1 (MP4 11380 KB)

Supplementary file2 (MP4 9413 KB)

Supplementary file3 (MP4 23173 KB)

Supplementary file4 (MP4 7341 KB)

Supplementary file5 (MP4 5707 KB)

Supplementary file6 (MP4 5706 KB)

Supplementary file7 (MP4 4616 KB)

Supplementary file8 (MP4 9365 KB)

Supplementary file9 (MP4 3608 KB)

Supplementary file10 (PDF 1413 KB)
