# Supplementary material for: Biodegradable, Super-Strong, and Conductive Cellulose Macrofibers for Fabric-Based Triboelectric Nanogenerator
Source: Nanomicro Lett. 2022 Apr 28;14:115. doi: 10.1007/s40820-022-00858-w (PMC9050994; doi:10.1007/s40820-022-00858-w)
Supplement: Supplementary file 10 — Supplementary file10 (PDF 1413 KB) [file 40820_2022_858_MOESM10_ESM.pdf]

Supporting Information for

Biodegradable, Super-Strong and Conductive Cellulose Macrofibers for Fabric-Based Triboelectric Nanogenerator

Sanming Hu[1, 2, 6, #], Jing Han[2, 3, #], Zhijun Shi[1, 6, #], Kun Chen[1], Nuo Xu[2, 3], Yifei Wang[2, 3], Ruizhu Zheng[1], Yongzhen Tao[6], Qijun Sun[2, 3, 4, *], Zhong Lin Wang[2, 3, 5, *], Guang Yang[1, *]

[1]College of Life Science and Technology, Huazhong University of Science and Technology, Wuhan, 430074, P. R. China

[2]Beijing Institute of Nanoenergy and Nanosystems, Chinese Academy of Sciences, Beijing 101400, P. R. China

[3]School of Nanoscience and Technology, University of Chinese Academy of Sciences, Beijing 100049, P. R. China

[4]Center on Nanoenergy Research, School of Physical Science and Technology, Guangxi University, Nanning 530004, P. R. China

[5]School of Materials Science and Engineering, Georgia Institute of Technology, Atlanta, Georgia 30332-0245, United States

[6]State Key Laboratory of New Textile Materials and Advanced Processing Technologies, Wuhan Textile University, Wuhan 430200, P. R. China

[#]Sanming Hu, Jing Han and Zhijun Shi contributed equally to this work.

*Corresponding authors. E-mail: sunqijun@binn.cas.cn (Qijun Sun); zhong.wang@mse.gatech.edu (Zhong Lin Wang); gyang-hust@hust.edu.cn (Guang Yang)

Supplementary Figures and Tables

Supplementary Figures

Figure S1 The schematic illustration of preparation process of BC/CNT and BC/CNT/PPy hydrogels

Figure S2 SEM images of unstretched and stretched hydrogels of pure BC, BC/CNT, BC/CNT/PPy

Figure S3 SEM and TEM images of CNTs and PPy

Figure S4 Synthesis mechanism of PPy with P-TSA as a dopant

Figure S5 SEM of PPy particles and corresponding EDS elements

Figure S6 Fracture SEM of BC/CNT/PPy macrofibers and corresponding EDS elements

Figure S7 The charging curve of the 100 μF by pressing the fabric-based TENG at 4 Hz

Figure S8 The air permeability of woven cellulose-base/nylon macrofiber fabric

Figure S9 The energy-generating mechanism of the fabric-based TENG

Figure S10 Relationship between current and voltage of the external load

Figure S11 The charging curve of the 100 μF by pressing the fabric-based TENG at 4 Hz

Supplementary Tables

Table S1 Summary and comparison of fabric-based TENGs based various electrodes

Table S2 Summary of degradable or green materials for fabricating TENG

Table S3 Elementary Analysis of BC/CNT macrofibers, CNT content, Electrical Conductivity, Tensile strength and Young's modulus

Table S4 Elementary Analysis of BC/CNT/PPy macrofibers, CNT content, Electrical Conductivity, Tensile strength and Young's modulus.

Supplementary Movies

Movie S1 The fabric-based TENG powers a watch

Movie S2 The fabric-based TENG powers a temperature-humidity meter

Movie S3 The fabric-based TENG powers a calculator

Movie S4 The fabric-based TENG as self-powered sensor monitors the walking of human

Movie S5 The fabric-based TENG as self-powered sensor monitors the running of human

Movie S6 The fabric-based TENG as self-powered sensor monitors the jumping of human

Movie S7 The fabric-based TENG as self-powered sensor monitors the raising arm of human

Movie S8 The fabric-based TENG as self-powered sensor monitors the bending arm of human

Movie S9 The fabric-based TENG as self-powered sensor monitors the lifting leg of human

Table S1. Summary and comparison of fabric-based TENGs based various

Mode	Electrodes	Triboelectric materials	Structures	Power outputs	Stretchability/durability	Washability	Applications	Ref.
SE	Cu fiber	Silicone and polyester	Nanofiber	25 V, 12.8 nA (40 Hz)	20000 cycles	60 min	Sleep monitoring	13
CS	Ni coating	Cotton and parylene	2D weaving	40 V, 5 μA, 2.42 mW cm^{-1} (5 Hz)	10000 cycles	-	Energy harvesting	14
FT	Ag fabric	Skin and mxene/silicon	2D weaving	1.34 kV, 55 W m^{-2}	86000 cycles	-	Energy harvesting	15
CS	Al foil	Nylon fabric and PVDF	Fabric stacking	2 V, 200 nA	Durable	50 times	Pulse signal monitoring	16
SE	Stainless-steel rod	Silicone rubber and polyester	2D weaving	10.79 Mv/Pa	20000 cycles	Washable	Sleep monitoring	17
SE	Au foil	Water drop, waterproof fabric	Fabric stacking	15 V, 4 μA, 0.14 W m^{-2}, (100 MΩ)	Stretchable and durable	Waterproof	Water energy	18
CS	Ag yarn	PA66 and PTFE	Fabric stacking	50 V, 0.9 μA, 7.531 W m-2 (40 MΩ)	45%, 6000cycles	-	Energy harvesting	19
SE	CNT coating	Skin fabric and skin	Fabric stacking	7 V, 180 nA (2 Hz)	2000 bending	Washable	Touch sensor	20
CS	Graphene coating	PTFE and nylon	3D spacer	3 V, 0.3 μA, 16 μW (0.6 MΩ)	-	-	Pressure sensing	21
FT	Carbon fabric	PI/PU and AI/PDMS	Lateral sliding	15 V, 140 nA (3 cm s^{-1})	Durable	-	Energy harvesting	22
CS	CNTcoating	Cotton thread and PTFE	2D weaving	12.5 V, 11.22 nA, 11.08 Nw (100 MΩ)	2.15%	-	Motion sensor	23
CS	PPy coating	PDMS and cotton	Fabric stacking	200 V, 6 μA, 82 μW cm^{-2}	5000 cycles	-	Energy harvesting	24
CS	PEDOT:PSS	PEDOT:PSS and silicone rubber	Multiarch	0.2 V(160% strain)	160%/4 h	Washable	Wearable keyboard	25
SE	PEDOT:PSS coating	PEDOT:PSS and PFTE	Fabric stacking	49.7 V, 787 nA, 2.67 Mw (9 MΩ)	160%	Washable	multi-arch strain sensor	26
SE	KI-Gly	Silicone rubber and Acylic	Lateral sliding	300 V, 17.5 Mw m-2	250%		Motion sensing	27
SE	Galinstan liquid electrode	Silicone rubber skin	2D wearing	130 V ,4 μA (2Hz)	300%	-	Wearable electronics	28
SE	Ag NWs/CNT	Ag NWs/CNT/PDMS	Coaxial structure	22 V, 7.5 nC, 21.516 μW (150 MΩ)	1800 times	-	Tactile sensor and motion sensor	29

SE:single electrode; CS: contact-separation; FT: freestanding triboelectric-

Table S2. Degradable or green materials for fabricating TENG

Triboelectric Materials	Preparing method	Electrodes	Output performance	Durability	Degradation time	Applications	Ref.
PLGA film	Electrospining	Ag nanowires	97 V, 1.5 µA, 130 mW m^{-2}	50000 times	-	E-skin, monitoring of physiological signal	33
Starch flim	Casting	Al film	2.1V	4600 cycles	15 days	Energy harvesting	34
Diatom-CNF film	Vacuum filtration	Al tapes	380 V, 18 µA, 80 mW m^{-2} (5 Hz)	-	-	Energy harvesting, breath sensing	35
Laver film	Vacuum filtration	Ag leaf	23 V, 315 nA 0.8 mW m^{-2}	1000 cycles	28 days	Energy harvesting	36
Chitosan based film	Casting	Al film	13.5 V, 42 nA, 17.5 µW m^{-2} (5 Hz)	8400 times	15 min	-	37
Rice paper	Sticking	Conductive ink	392 V, 16.7 µA 82.69µW m^{-2} (5 Hz)	10000 cycles	-	Energy harvesting	38
Silk nanofiber membrane	Electrospining	Al film	18 V 4.3 mW m^{-2} (5 Hz)	25000 cycles	-	Energy harvesting	39
BC film	Bacterial synthesis	BC composite	29 V, 0.6 µA, 0.08 µW (5 Hz)	10000 cycles	7 hours	Energy harvesting	40
Plant protein film	Casting	PEDOT:PSS gel	20.3 V, 1.26 µA	40000 cycles	127 days	Self-powered interface mulch film for crop growth	41
PLA and gelatin	Electrospining and casting	Mg electrode	500 V, 5 W m^2	13500 cycles	20 days	Energy harvesting	42
BC macrofiber	Wet-stretching and twisting	BC composite macrofiber, Al membrane	170 V, 7.5µA, 54.14 mW m-2 (5 Hz)	1000 cycles	108 hours	Energy harvesting, motion monitoring	Ours

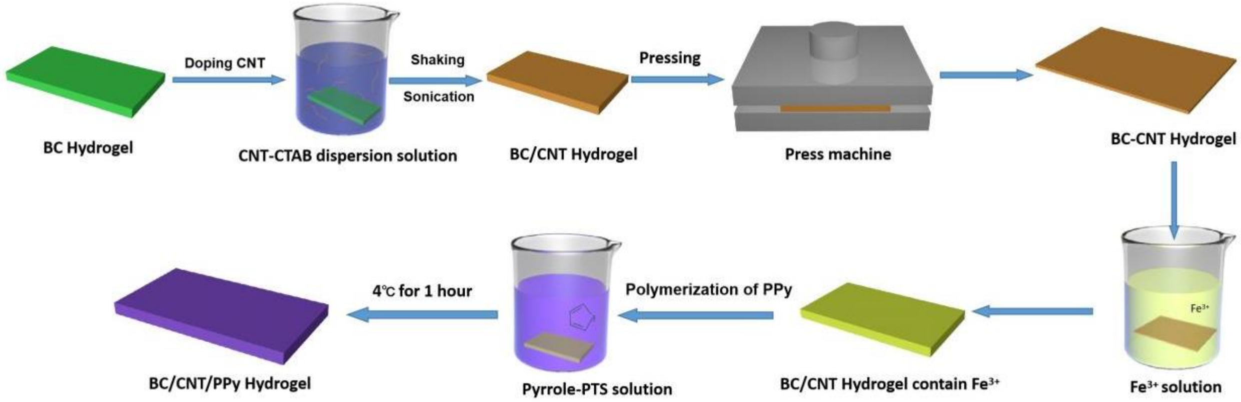

Figure S1. The schematic illustration of preparation process of BC/CNT and BC/CNT/PPy hydrogels.

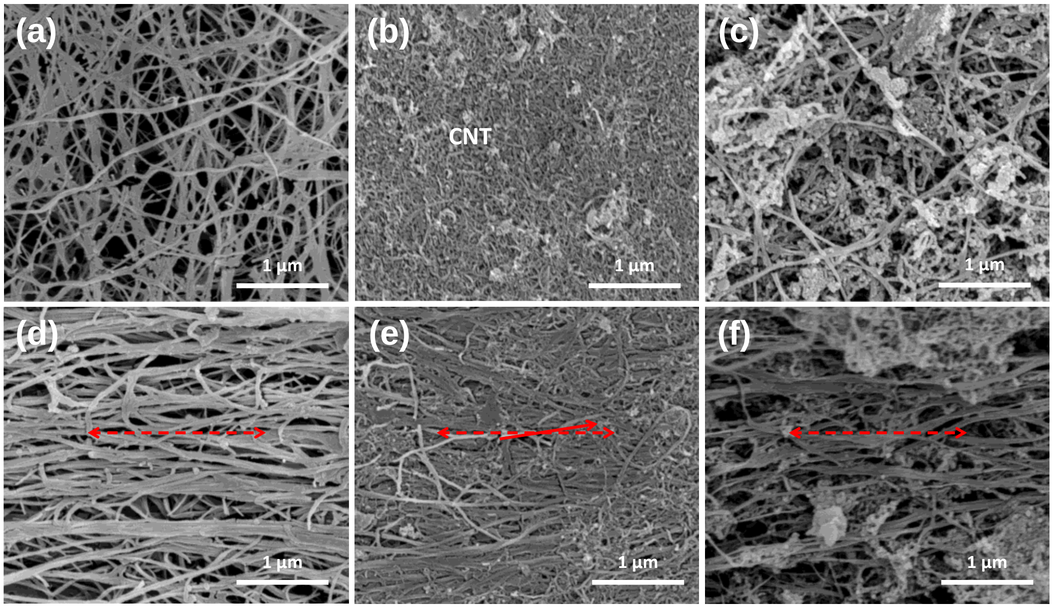

Figure S2. SEM images of pure BC, BC/CNT, BC/CNT/PPy hydrogel. The surface morphology of (a) BC, (b) BC/CNT, (c) BC/CNT/PPy, (d) stretched BC, (e) stretched BC/CNT, (f) stretched BC/CNT/PPy.

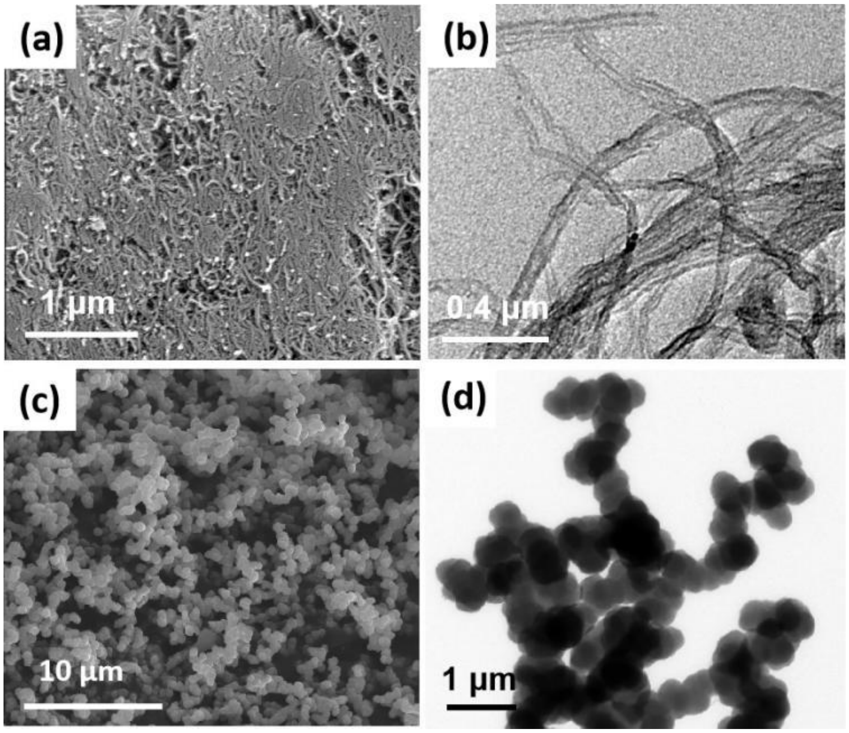

Figure S3. SEM images of (a) CNTs, (c) PPy. TEM images of (b) CNTs, (d) PPy.

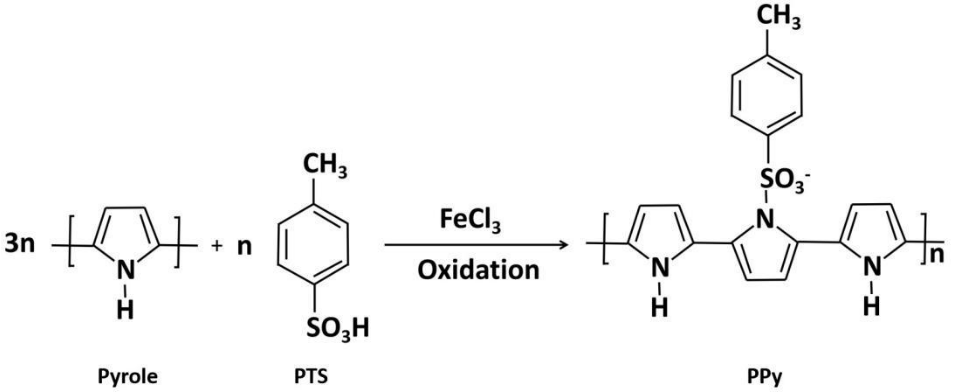

Figure S4. Synthesis mechanism of PPy with P-TSA as a dopant.

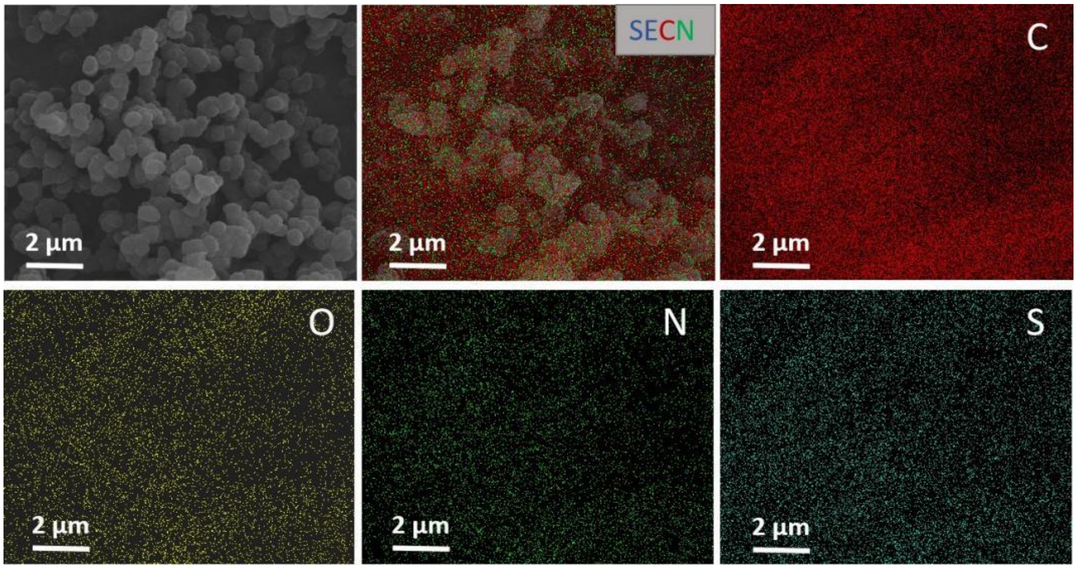

Figure S5. SEM of PPy particles and corresponding EDS elements are mapping for C, N, O, and S.

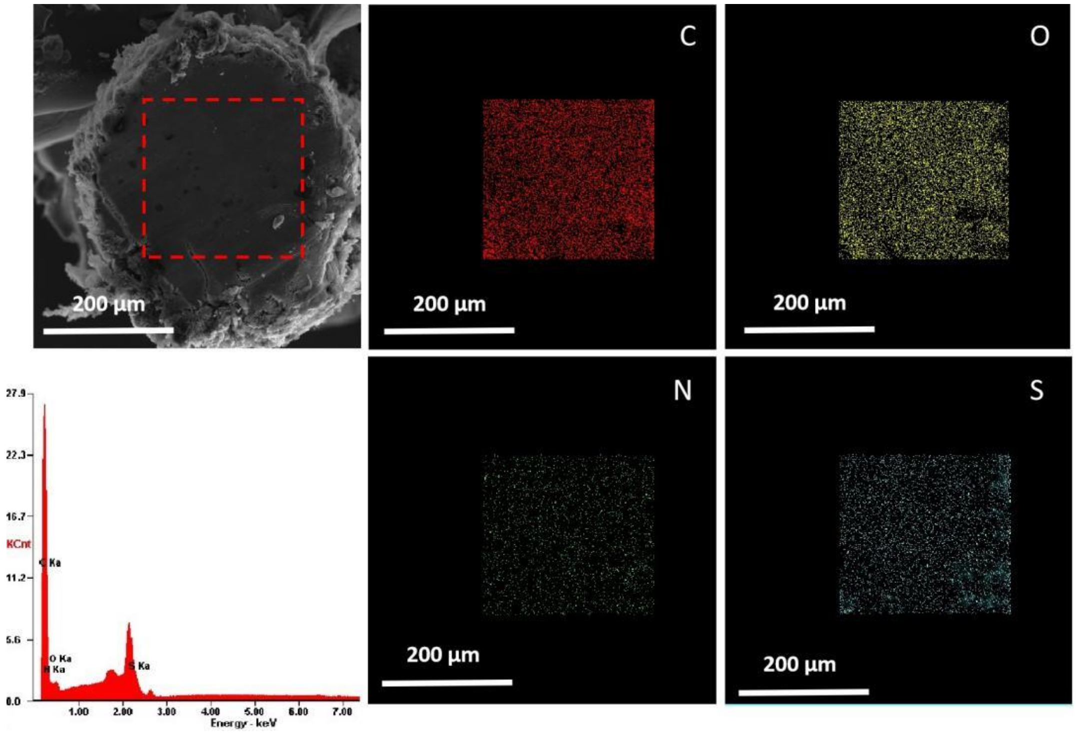

Figure S6. Fracture SEM of BC/CNT/PPy macrofibers and corresponding mapping for C, N, O, and S.

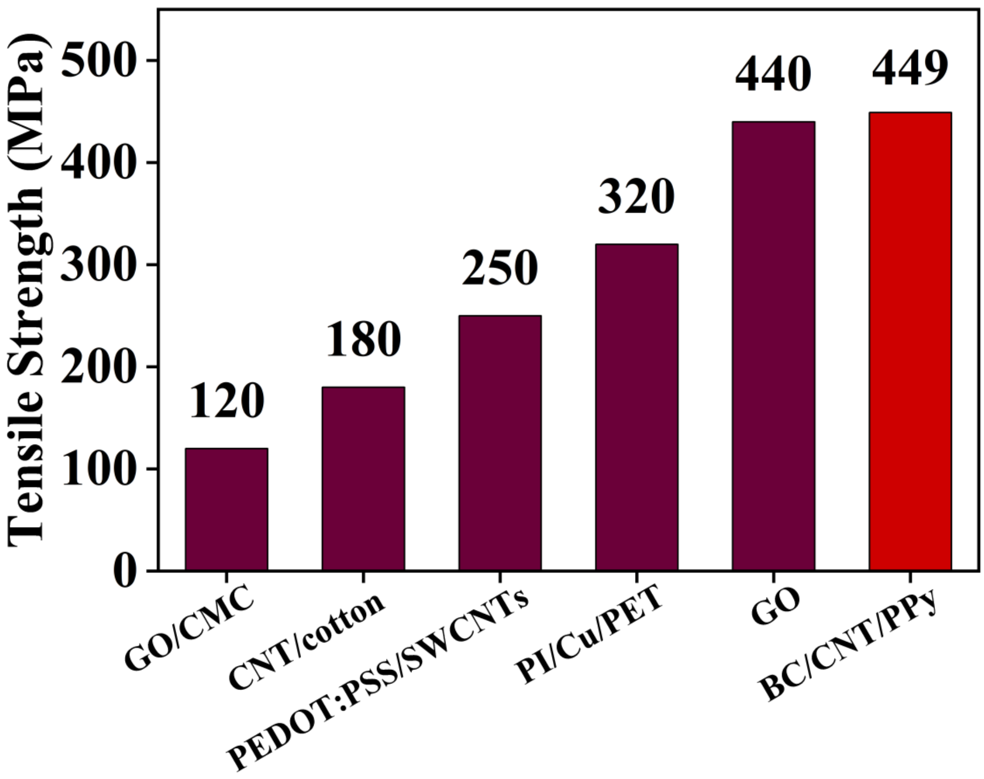

Figure S7. Comparison of tensiles strength of BC/CNT/PPy conductive macrofiber and other conductive macrofibers.

Table S3. Elementary Analysis of BC/CNT macrofibers, CNT content, Electrical Conductivity, Tensile strength and Young's modulus.

Macrofibers	CNT content (wt%)	C (%)	H (%)	N (%)	CNT content (wt%)	Electrical Conductivity (S/cm)	Tensile strength (MPa)	Young's modulus (GPa)
BC		42.47	5.96	0.02	0	2E-13	825.7 ± 42.6	52.2 ± 0.92
BC/CNT-1	0.025	43.23	6.14	0.00	2.36	0.65 ± 0.04	680.8 ± 47.1	45 ± 3.64
BC/CNT-2	0.05	43.64	6.16	0.01	3.06	0.92 ± 0.03	671.4 ± 93	42.7 ± 4.26
BC/CNT-3	0.1	44.10	6.13	0.01	3.86	1.59 ± 0.01	549.1 ± 40	28 ± 1.32
BC/CNT-4	0.2	46.01	6.06	0.06	7.14	2.22 ± 0.12	487.1 ± 32	25.6 ± 0.8
BC/CNT-5	0.3	46.8	6.04	0.05	8.5	1.72 ± 0.09	376.3 ± 55.7	12.5 ± 1.88

Table S4. Elementary Analysis of BC/CNT/PPy macrofibers, PPy content, Electrical Conductivity, Tensile strength and Young's modulus.

Macrofibers	Conc of pyrrole (mol)	C (%)	N (%)	S (%)	PPy Content (wt%)	Electrical Conductivity (S/cm)	Tensile strength (MPa)	Young's modulus (GPa)
BC/CNT-4		46.01	0.06	0.00	0	2.22 ± 0.12	487.1 ± 32	25.6 ± 0.8
BC/CNT/PPy-1	0.01	44.04	1.00	0.27	3.03	2.53 ± 0.06	450.4 ± 14.7	35.6 ± 2.8
BC/CNT/PPy-2	0.03	47.84	3.27	1.13	12.69	3.77 ± 0.24	515.9 ± 74.8	29.3 ± 0.08
BC/CNT/PPy-3	0.05	49.11	4.44	2.06	23.14	5.32 ± 0.12	448.4 ± 29.3	19 ± 5.95
BC/CNT/PPy-4	0.07	51.11	5.51	2.67	30	5.02 ± 0.37	317.1 ± 21.3	13.8 ± 2.24
BC/CNT/PPy-5	0.09	48.58	4.92	2.38	26.74	5.45 ± 0.4	136.3 ± 26.3	5.1 ± 0.03

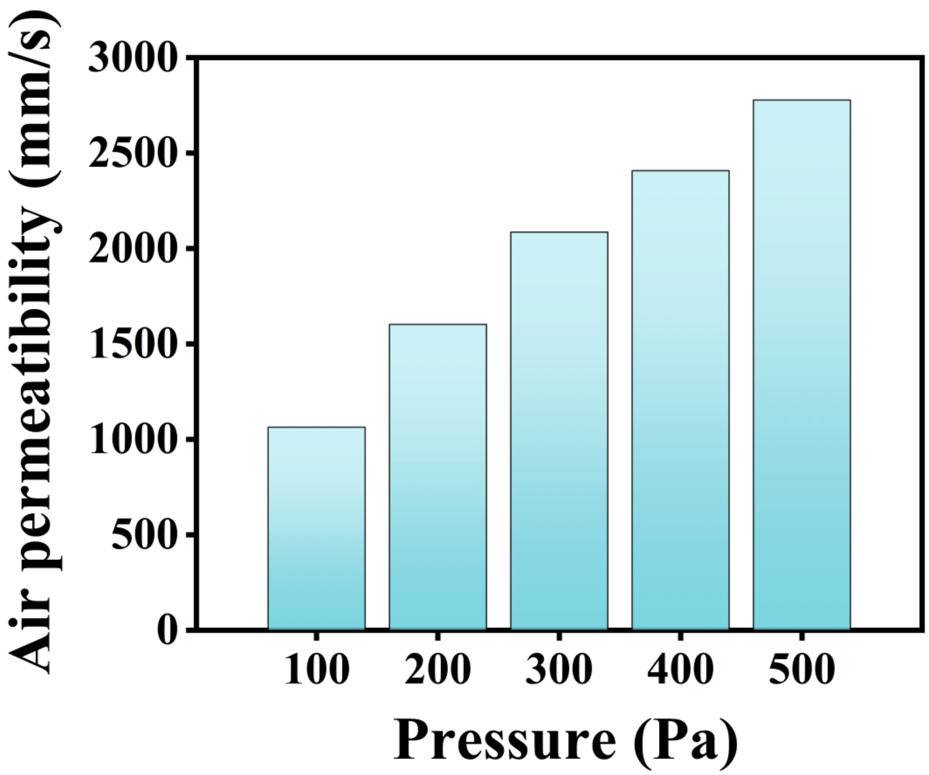

Figure S8. The air permeability of fabric-based TENG.

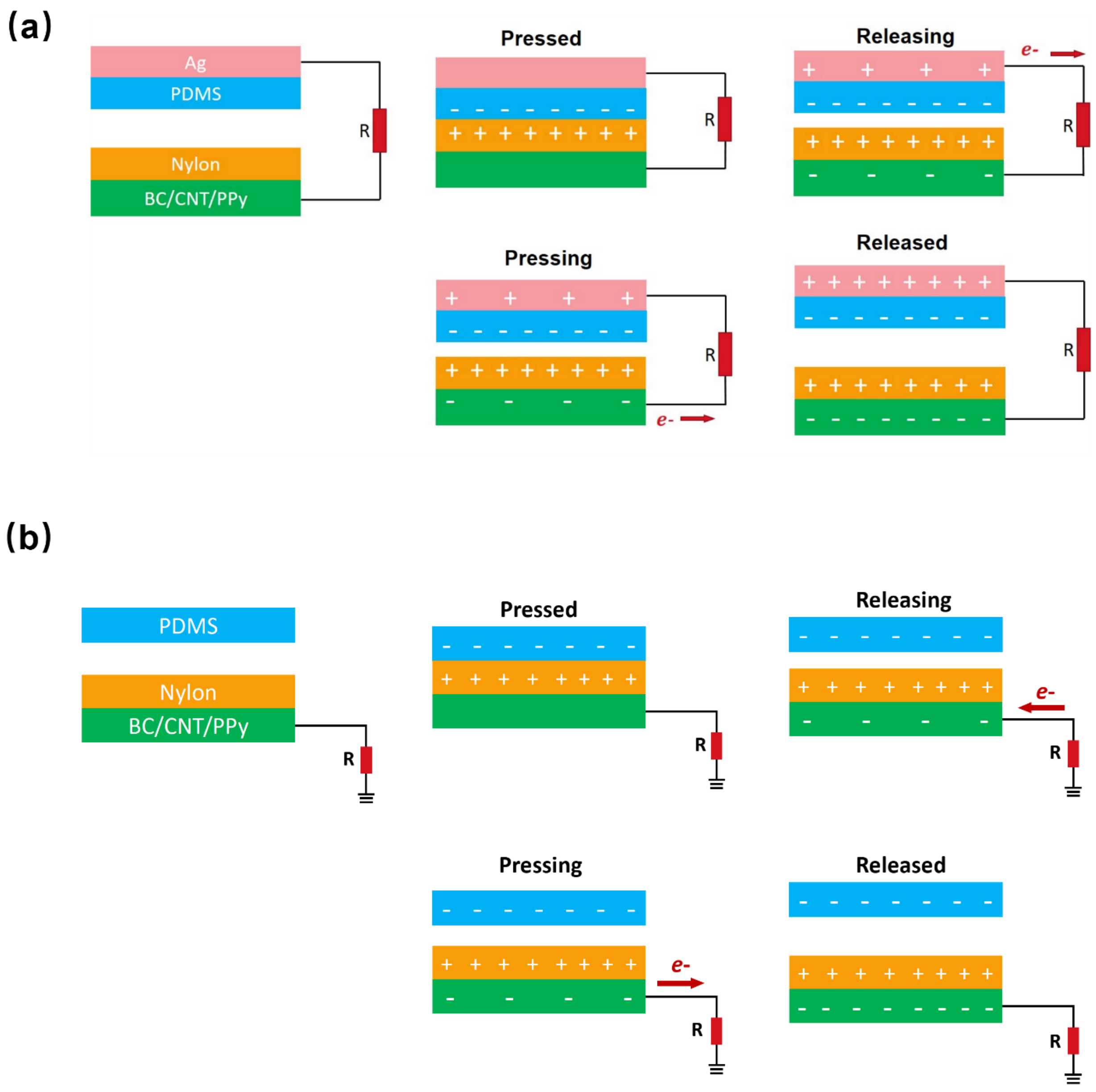

Figure S9. The energy-generating mechanism of the fabric-based TENG, (a) contact-separation mode and (b) single electrode mode.

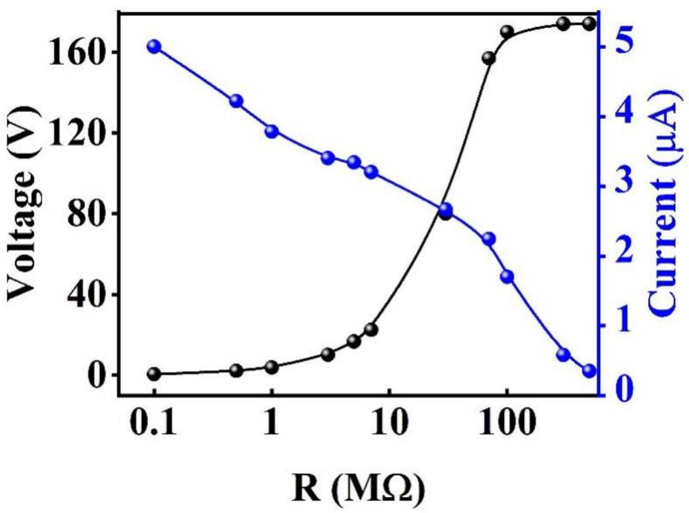

Figure S10. Relationship between current and voltage of the external load.

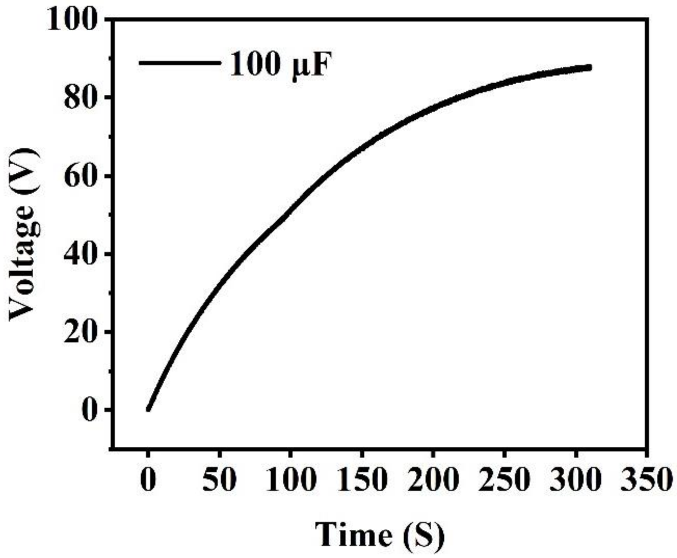

Figure S11. The charging curve of the 100 μF by pressing the fabric-based TENG at 4 Hz.
